# Proteomic Alterations and Oxidative Stress in Seminal Plasma of Nellore Bulls Under Sexual Rest

**DOI:** 10.3390/ijms26062457

**Published:** 2025-03-10

**Authors:** Ekaette Chris Udoekong, Camilo Jose Ramirez-Lopez, Denise Silva Okano, Edvaldo Barros, Pedro Marcus Pereira Vidigal, Iara Magalhães Ribeiro, Renner Philipe Rodrigues Carvalho, Mariana Machado-Neves, José Domingos Guimarães, Simone Eliza Facioni Guimarães

**Affiliations:** 1 Laboratory of Animal Biotechnology, Department of Animal Science, Universidade Federal de Viçosa, Viçosa 36570-900, Brazil; ekachris.udoekong@gmail.com (E.C.U.); sfacioni@ufv.br (S.E.F.G.); 2 Laboratory of Animal Reproduction, Department of Veterinary Medicine, Universidade Federal de Viçosa, Viçosa 36570-900, Brazil; deniseokano@gmail.com (D.S.O.); jdguima@ufv.br (J.D.G.); 3 Núcleo de Análise de Biomoléculas, Universidade Federal de Viçosa, Viçosa 36570-900, Brazil; edvaldo.barros@ufv.br (E.B.); pedro.vidigal@ufv.br (P.M.P.V.); 4 Laboratory of Structural Biology, Department of Biology, Universidade Federal de Viçosa, Viçosa 36570-900, Brazil; iara.m.ribeiro@ufv.br (I.M.R.); renner.carvalho@ufv.br (R.P.R.C.); mariana.mneves@ufv.br (M.M.-N.)

**Keywords:** sperm parameters, antioxidant enzymes, proteins, reproductive efficiency

## Abstract

Sexual rest (SR) in bulls leads to the accumulation of senescent spermatozoa in the extragonadal reserves, potentially affecting semen quality and reproductive efficiency. Therefore, this study aimed to investigate the impact of SR on the seminal plasma proteome and oxidative status of Nellore bulls. Six adult bulls were subjected to 195 days of SR and sequential semen collections using the electroejaculation method. The ejaculates were analyzed to assess sperm quality. Seminal plasma from the first and last ejaculates was evaluated for oxidative status and proteomic profile using LC-MS. The results revealed significant improvements in sperm motility, vigor, and antioxidant enzyme activity (superoxide dismutase and catalase) in the last ejaculate compared to the first. Conversely, higher levels of oxidative markers, such as malondialdehyde and carbonyl proteins, were observed in the first ejaculate. Proteomic analysis identified 156 proteins, with 28 differentially abundant between ejaculates. The first ejaculate showed a higher abundance of proteins linked to acrosomal exocytosis and energy metabolism, while proteins associated with sperm motility and immune modulation were elevated in the last ejaculate. These findings suggest that SR induces oxidative stress and proteomic alterations in seminal plasma, negatively affecting sperm quality, emphasizing the need for strategic reproductive management in bulls.

## 1. Introduction

Cattle farming plays a central role in the Brazilian economy, contributing significantly to the Gross Domestic Product (GDP) of agribusiness and positioning the country as one of the largest producers and exporters of beef globally [1,2]. The sustainability and growth of this sector directly depend on herds’ reproductive efficiency, a major challenge faced by the industry [3]. Among the critical factors influencing this efficiency, bull fertility and semen quality are crucial, as they directly impact pregnancy rates and the success of assisted reproduction programs [4,5].

Reproductive efficiency in livestock is not solely a matter of genetics; it arises from a complex interplay of genetic, environmental, management, and health factors [6,7,8]. In this context, selecting bulls with high fertility and adaptability to local environmental conditions is essential for ensuring the economic viability of production systems [9]. However, bull fertility can be compromised by various factors, with sexual rest (SR), being particularly impactful, especially in natural breeding systems [10]. Characterized by prolonged periods of sexual inactivity, SR can significantly affect semen quality, thereby reducing bull fertility and herd reproductive efficiency [11].

Although SR is a natural and reversible physiological state, it leads to the accumulation of lower-quality sperm in extragonadal ducts, such as the cauda epididymis and vas deferens ampullae [12]. This accumulation results in ejaculates with high volume, low motility, and a high percentage of defective sperm [13]. These factors can severely compromise pregnancy rates and overall reproductive efficiency in commercial systems. Moreover, a lack of awareness of the typical changes in sperm parameters during SR can lead to the premature culling of bulls that could otherwise be productive under normal conditions.

The etiology of SR is not entirely clear, and the accumulation of spermatozoa may be attributed to failures in the innervation mechanisms of the smooth muscle fibers in the cauda epididymis and the vas deferens, which are responsible for the contractions that facilitate sperm transport [14,15,16]. Alterations in the pH of the epididymal luminal microenvironment could also increase the metabolic rate of spermatozoa and promote the development of an oxidative state [17].

Spermatozoa are particularly sensitive to oxidative stress-induced alterations due to their reduced cytosol, high concentration of polyunsaturated fatty acids in their plasma membrane, and limited antioxidant defense system [18]. In this context, the highly specialized reactive oxygen species scavenging system of seminal plasma provides antioxidant protection to spermatozoa [19].

Seminal plasma is a complex fluid derived from male accessory glands, including the seminal vesicles, prostate, bulbourethral glands, and epididymis, and it plays a key role in the maintenance of sperm function [20]. Its proteins are involved in fundamental processes such as sperm capacitation, motility, sperm–oocyte fusion, and early embryonic development [21,22]. During SR, changes in the seminal plasma proteome may reflect modifications in sperm biology, including increased oxidative stress and the activation of pathways detrimental to fertility [13,23]. These molecular changes, often linked to sperm aging, can reduce sperms’ viability and functionality [24].

Proteomic analysis has emerged as a powerful tool to investigate the molecular mechanisms underlying sperm function and fertility. The characterization of the seminal plasma proteome enables the identification of key proteins involved in sperm viability, motility, and the oxidative stress response, providing insights into the physiological and pathological processes affecting male fertility [25,26,27]. Studies have shown that oxidative stress alters the seminal plasma proteome, leading to modifications in protein abundance and function, which can ultimately compromise sperm quality [28,29].

Given that oxidative stress is a major consequence of prolonged sperm storage in extragonadal reserves, significant molecular changes are expected in seminal plasma proteins. In this scenario, a proteomic approach is essential to identifying specific alterations that may compromise sperm function and fertility after SR. By investigating these molecular changes, it is possible to determine how oxidative stress impacts key proteins involved in sperm physiology, helping to elucidate the mechanisms responsible for reduced fertility after prolonged sexual inactivity.

Considering the vital role of seminal plasma proteins in regulating fertility, understanding how SR affects the seminal plasma proteome is essential to elucidate the mechanisms behind the reduced fertility observed in resting bulls. This study aims to investigate the impact of SR on the seminal plasma proteome and oxidative status of Nellore bulls and evaluate the implications of these alterations on sperm quality and reproductive efficiency. Understanding these molecular alterations is crucial for optimizing reproductive management strategies, improving semen evaluation protocols, and ensuring the selection of bulls with a higher fertility potential.

Our findings highlight that SR induces oxidative stress and proteomic changes in seminal plasma, negatively affecting sperm function, which emphasizes the need for strategic reproductive management post-SR.

## 2. Results

### 2.1. Sperm Quality Analysis

**Figure 1:**
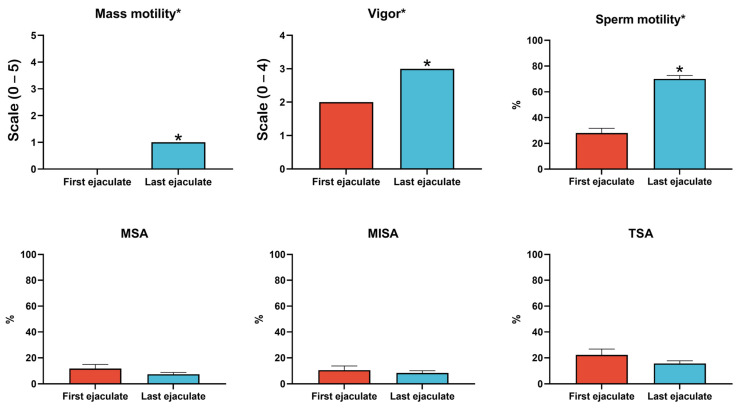
Comparison of sperm quality parameters in Nellore bulls after prolonged SR and during resumed sexual activity. Significant improvements were observed in mass motility, sperm motility, and vigor in the last ejaculate, following the resumption of sexual activity, compared to the first ejaculate collected immediately after 195 days of SR (*p* < 0.05). These findings reflect the recovery of sperms’ functionality after clearing senescent spermatozoa from the extragonadal reserves. No significant differences were observed in major (MSA), minor (MISA), or total sperm abnormalities (TSA) between the ejaculates. Asterisks indicate significant differences between ejaculates (*p* < 0.05). Error bars represent the standard error (SE).

Four semen collections per bull were required to remove the senescent sperm populations that had accumulated in the extragonadal reserves during SR and to restore sperm parameters in accordance with Brazilian College of Animal Reproduction (CBRA) guidelines. Significant differences (*p* < 0.05) in sperm quality characteristics, including mass motility, sperm motility, and vigor, were observed between the first and last ejaculates, with higher values recorded in the last ejaculates (Figure 1). However, the percentages of major, minor, and total sperm abnormalities did not differ significantly (*p* > 0.05) between the ejaculates.

### 2.2. Oxidative Stress Analysis

**Figure 2:**
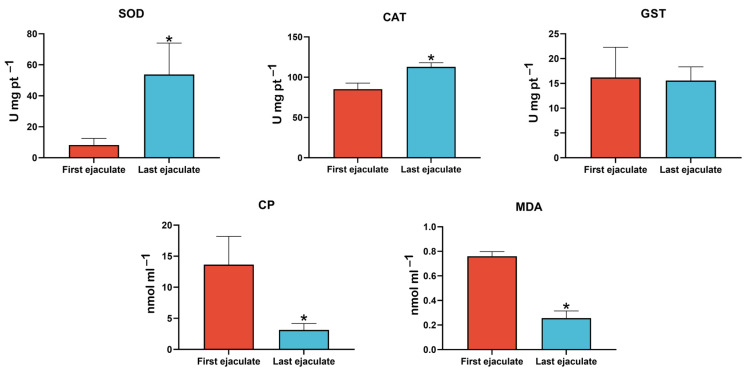
Antioxidant enzyme activity and oxidative stress markers in seminal plasma from Nellore bulls after SR and during resumed sexual activity. Seminal plasma from the first ejaculate exhibited significantly lower activities of superoxide dismutase (SOD) and catalase (CAT), indicating diminished antioxidant defenses due to prolonged sperm storage. In contrast, these enzyme activities were significantly higher in the last ejaculation following resumed sexual activity (*p* < 0.05). Elevated levels of oxidative markers, such as malondialdehyde (MDA) and carbonyl proteins (CPs), were observed in the first ejaculate, reflecting increased oxidative damage. Glutathione S-transferase (GST) activity showed no significant differences between the ejaculates. Asterisks indicate significant differences between ejaculates (*p* < 0.05). Error bars represent the standard error (SE).

The activity of antioxidant enzymes and the concentration of oxidative markers in seminal plasma were compared between the first and last ejaculates of sexually rested bulls (Figure 2). The results revealed that superoxide dismutase (SOD) and catalase (CAT) activity was significantly lower in the seminal plasma of the first ejaculate compared to the last (*p* < 0.05). This reduction suggests a decrease in the initial antioxidant capacity associated with prolonged sperm storage. Conversely, the activity of Glutathione S-transferase (GST) did not show significant differences between the ejaculates. Regarding oxidative markers, higher concentrations of malondialdehyde (MDA) and carbonyl proteins (CPs) were observed in the first ejaculate compared to the last (*p* < 0.05). These findings indicate increased lipid peroxidation and oxidative damage to proteins in the first ejaculate. * *p* < 0.05.

### 2.3. Qualitative Proteomics Analysis

**Figure 3:**
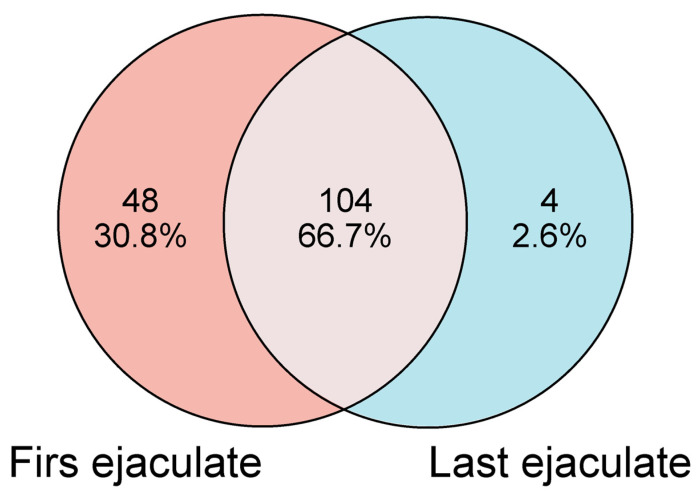
The Venn diagram illustrates the distribution of proteins identified in seminal plasma from Nellore bulls after SR and during resumed sexual activity. A total of 156 proteins were identified, with 104 proteins common to both the first ejaculate, collected immediately after SR, and the last ejaculate, obtained after sexual activity resumed. Forty-eight proteins were unique to the first ejaculate, and four were unique to the last ejaculate.

An LC-MS data analysis identified 156 proteins in the seminal plasma from SR Nellore bulls (Table S1). Of these, 152 proteins were detected in the seminal plasma of the first ejaculate (Table S2), whereas 108 proteins were identified in the last ejaculate (Table S3). A comparative analysis of the proteomic profiles between the first and last ejaculates revealed that 104 proteins (66.7%) were present in both samples. However, 48 proteins (30.8%) were specifically detected in the seminal plasma of the first ejaculate, while only 4 proteins (2.6%) were unique to the last ejaculate (Figure 3).

**Figure 4:**
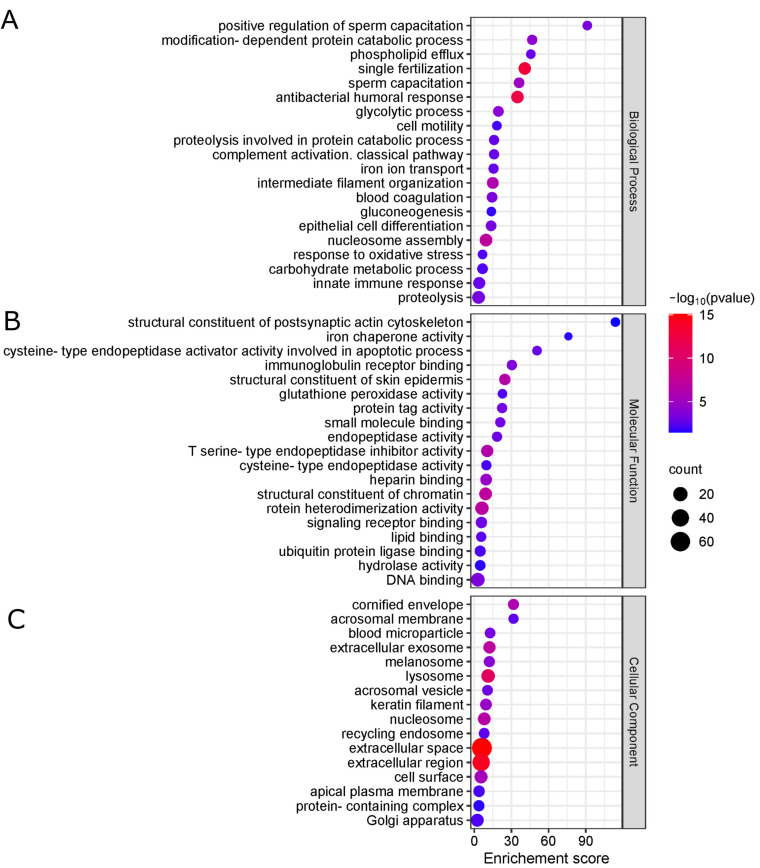
Gene Ontology (GO) analysis of seminal plasma proteins from Nellore bulls after SR and during resumed sexual activity. (**A**) Biological processes: Proteins from the first ejaculate were enriched in processes related to oxidative stress and acrosomal exocytosis, while proteins from the last ejaculate were associated with sperm motility and fertilization. (**B**) Molecular functions: The identified proteins were involved in antioxidant activity, cytoskeletal structure, and immunoglobulin binding, reflecting functional shifts between oxidative stress during rest and recovery after resumed sexual activity. (**C**) Cellular components: Proteins were predominantly localized in the acrosomal membrane, extracellular exosomes, and cell surface, illustrating dynamic changes in sperm membrane composition and function.

Based on gene ontology analysis, the main biological processes associated with the proteins in the seminal plasma of bulls in SR include the positive regulation of sperm capacitation, protein catabolism dependent on post-translational modification, phospholipid efflux, and single fertilization (Figure 4A). Regarding molecular function, the proteins were predominantly associated with the structural constitution of the cytoskeleton, iron chaperone activity, cysteine-type endopeptidase activity, and immunoglobulin binding, in addition to antioxidant activity, particularly glutathione peroxidase activity (Figure 4B). As for the cellular component, the proteins were primarily located in the acrosomal membrane, extracellular exosomes, extracellular space, and cell surface (Figure 4C).

The enrichment analysis of signaling pathways in proteins identified in the seminal plasma of bulls during SR revealed that the lysosome-related pathway was the most significantly enriched. This suggests the heightened degradation and recycling activity of cellular components in the seminal plasma during this period. Additionally, the apoptosis signaling pathway showed a significant level of enrichment, indicating the presence of proteins associated with programmed cell death. Other pathways related to immune response and inflammation, such as those involved in systemic lupus erythematosus and Staphylococcus aureus infection, were also enriched, underscoring the role of seminal plasma proteins in immune defense mechanisms.

**Figure 5:**
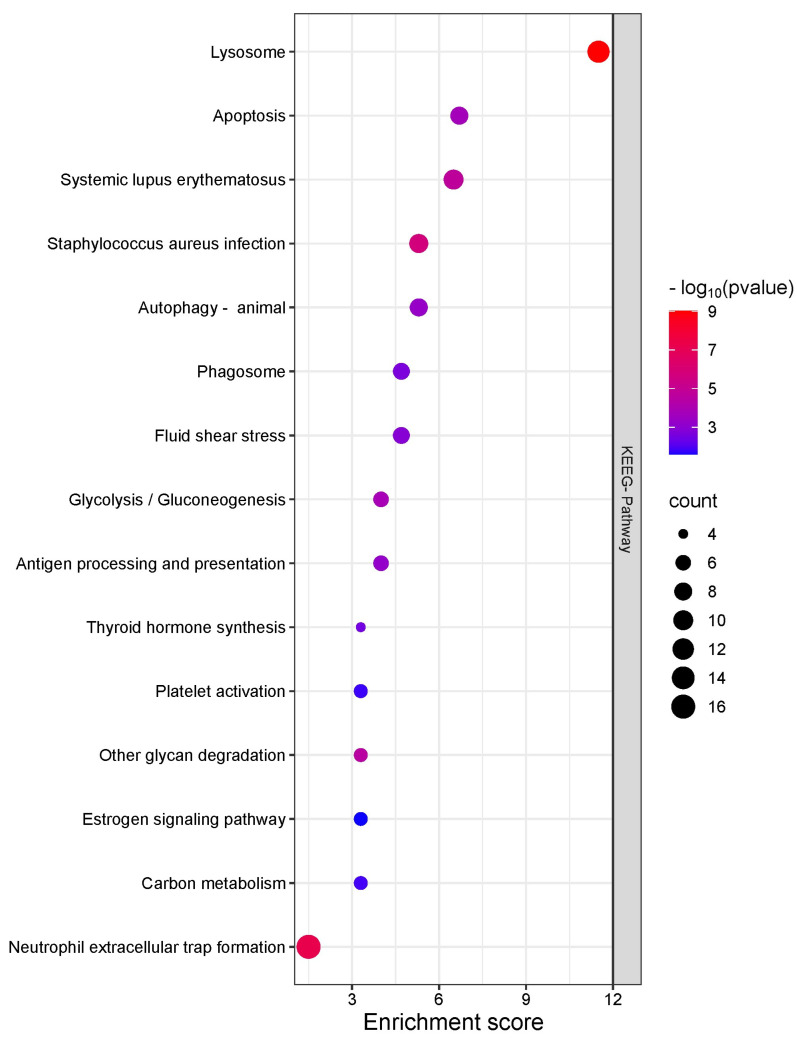
KEGG pathway enrichment analysis of seminal plasma proteins in Nellore bulls after SR and during resumed sexual activity. The first ejaculate, collected after prolonged SR, showed enrichment in lysosomal and apoptosis pathways—indicating increased protein degradation and cellular stress from sperm storage. Conversely, the last ejaculate demonstrated enrichment in pathways related to energy metabolism (glycolysis and gluconeogenesis) and immune modulation, consistent with improved sperm functionality and motility following the resumption of sexual activity.

Pathways involved in autophagy and phagosome formation, critical for maintaining cellular integrity and defending against pathogens, were also notably enriched. Moreover, the presence of proteins associated with glycolysis and gluconeogenesis was observed, reinforcing the central role of seminal plasma in sperm energy metabolism, even during periods of SR (Figure 5).

### 2.4. Quantitative Proteomics Analysis

**Figure 6:**
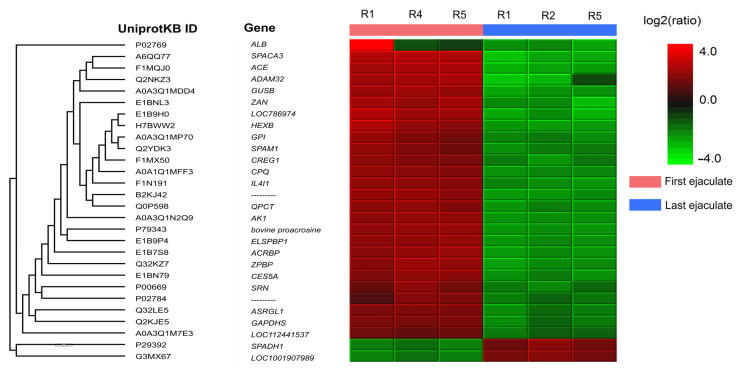
Differentially abundant proteins (DAPs) in seminal plasma from Nellore bulls after SR and during resumed sexual activity. A total of 28 proteins exhibited significant differences in abundance between the first ejaculate, collected post-SR, and the last ejaculate, collected after sexual activity resumed (fold change > 2.0, *p* < 0.01). The first ejaculate showed the upregulation of 26 proteins associated with oxidative stress, acrosomal exocytosis, and energy metabolism, reflecting cellular damage from prolonged sperm storage. In contrast, the last ejaculate exhibited an increased abundance of proteins linked to sperm motility, immune modulation, and fertilization, indicating restored reproductive function.

The principal component analysis of label-free quantification data validated the segregation among technical replicates of the experimental groups (Supplementary Figure S1). From the 150 seminal plasma proteins identified in both the first and last ejaculates, quantitative analysis revealed 28 differentially abundant proteins (DAPs) between the ejaculates. In the first ejaculate, 26 proteins were upregulated (Spermadhesin-1 and Protein LEG1 homolog), while in the last ejaculate, 26 proteins showed increased abundance (Figure 6). Proteins upregulated in the last ejaculate included ALB, ACE, CPQ, IL4I1, AK1, CES5A, ASRGL1, GAPDHS, LOC112441537, ZPBP, bovine proacrosin, SPACA3, ZAN, HEXB, GPI, ADAM32, GUSB, SPAM1, CREG1, SRN, ACRBP, ELSPBP1, and QPCT (Table S4). Of the 26 upregulated proteins in the first ejaculate, 7 (26.9%) proteins are related to the acrosome exocytosis process, and 7 (26.9%) proteins are related to energy metabolism.

## 3. Discussion

In this study, we evaluated the impact of SR on the proteomic profile and oxidative status of the seminal plasma of adult bulls of the Nellore breed and its relationship with changes in the physical characteristics of semen. Previous studies by our group demonstrated that SR alters the proteomic profile of spermatozoa and may be associated with premature triggering of the acrosomal reaction, potentially linked to oxidative stress [13]. Expanding on these findings, in the present study we observed that 26.9% of DAPs in the seminal plasma of the first ejaculate are related to the acrosomal exocytosis process. In addition, we identified a significant decrease in antioxidant enzyme activity and an increase in the concentration of oxidative markers in the first ejaculate, which reinforces the connection between oxidative stress and functional alterations in seminal plasma.

The effect of oxidative stress on sperm functionality was particularly evident when analyzing sperm quality parameters. The results of the sperm analysis indicate that SR negatively modifies the sperm profile of the breeders. However, these effects are reversible, since they do not affect spermatogenesis, as demonstrated by the differences observed in sperm quality parameters between the first and last ejaculate [25].

The prolonged storage of sperm in extragonadal reserves due to SR results in a decline in antioxidant enzyme activity, leading to excessive ROS accumulation. These reactive molecules cause extensive damage to chromatin, mitochondria, and plasma membranes, ultimately compromising sperm function and viability [26,27,28]. The results of the oxidative stress analysis reinforce this observation, because the first ejaculate presented a significantly lower activity of SOD and CAT compared to the last, accompanied by higher levels of MDA and CP, indicators of lipid peroxidation and protein oxidation, respectively.

The connection between oxidative stress and sperm quality has been widely documented in various species. Recent studies have shown a negative relationship between low SOD and CAT activity in seminal plasma and sperm parameters, semen freezability, and male fertility in boars, men, stallions, and buffaloes, respectively [29,30,31,32]. This relationship can be explained by the fundamental role that SOD and CAT play as the first line of antioxidant defense, catalyzing chemical reactions that eliminate superoxide anion and hydrogen peroxide, the main molecules responsible for lipid peroxidation in spermatozoa [33].

Beyond causing direct sperm damage, oxidative stress alters the seminal plasma microenvironment, leading to proteomic modifications that influence sperm function. In bovines, lipid peroxidation has been negatively correlated with sperm motility and fertilizing capacity, while also promoting plasma membrane lesions that can trigger premature acrosomal exocytosis. [34,35,36,37]. This scenario could explain the identification of DAPs such as Acrosin, Zonadhesin, Hyaluronidase, Zona Pellucida Binding Protein, and Sperm Acrosome Membrane-Associated Protein 3 in the seminal plasma of the first ejaculate. These proteins are constituents of the acrosomal matrix and play key roles in sperm–oocyte interaction, facilitating essential processes for fertilization [38,39,40]. The premature release of acrosomal proteins can compromise adequate fertilization and anticipate programmed sperm death [41,42].

Supporting this hypothesis, the KEGG pathway enrichment analysis highlights two key molecular pathways involved in sperm quality alterations: the lysosomal and apoptosis pathways. The lysosomal pathway could contribute to the degradation of damaged components derived from the peroxidation of polyunsaturated fatty acids in the sperm membrane and the recycling of oxidized proteins, essential functions in response to oxidative stress [43,44]. However, cellular aging associated with prolonged sperm storage and the accumulation of toxic waste could exceed the capacity of the lysosomes, promoting the release of enzymes such as cathepsins, which contribute to cell disruption and the activation of apoptotic pathways [45,46]. Indeed, a greater number of proteins from the cathepsin family were identified in the seminal plasma of the first ejaculate.

In a complementary manner, the apoptosis pathway, activated by oxidative stress, could explain the observed effects on sperm quality. The permeabilization of lysosomal and mitochondrial membranes, induced by ROS, facilitates the activation of caspase cascades that culminate in programmed cell death [47,48,49,50,51]. This mechanism not only affects senescent spermatozoa but could also contribute to alterations in the seminal microenvironment, aggravating the impact of SR on the proteomic profile of seminal plasma.

Glutathione peroxidase and albumin are proteins whose functions in seminal plasma are associated with antioxidant activity, due to their ability to reduce hydroperoxides and transport endogenous antioxidants such as ascorbic acid and uric acid [52,53]. Both proteins were identified as DAPs in the seminal plasma of the first ejaculate. Low glutathione peroxidase activity in seminal plasma has been related to low motility and high percentages of sperm abnormalities in donkeys and men, respectively [54,55]. However, we speculate that the high abundance of glutathione peroxidase and albumin in the seminal plasma of the first ejaculate may represent a compensatory local response to a pro-oxidant environment caused by the neutralization of SOD and CAT activity derived from the prolonged storage of spermatozoa in extragonadal reserves. In the meantime, specific tests should be performed to prove or refute our hypothesis.

Protein oxidation, a significant consequence of oxidative stress, plays a crucial role in sperm function. Elevated levels of ROS can lead to oxidative modifications, such as protein carbonylation, a non-enzymatic post-translational modification generated by the addition of carbonyl groups to the side chains of amino acids [56]. This modification is irreversible and stable and affects the function, folding, and hydrolysis of proteins [57]. Its expansion is favored by the formation of reactive aldehydes, such as malondialdehyde (MDA), which covalently bind to nearby proteins through Michael additions, introducing new carbonyl groups into their structures [58]. High concentrations of carbonylated proteins, such as those observed in the first ejaculate, have been identified in the semen of bulls, boars, and men with low sperm motility and reduced fertilizing capacity [59,60,61]. Additionally, Mostek et al. [62] reported that proteins with higher levels of carbonylation in the semen of bulls with low sperm quality were in the mitochondria and cytoskeleton, affecting energy generation and the flagellar machinery responsible for sperm motility.

In this study, seven DAPs in the seminal plasma of the first ejaculate are related to energy metabolism, including Glyceraldehyde-3-phosphate dehydrogenase, testis-specific (GAPDHS), a glycolytic enzyme that catalyzes the oxidative phosphorylation of Glyceraldehyde-3-phosphate to produce 1,3-diphosphoglycerate [63]. This compound is subsequently used by the enzyme phosphoglycerate kinase to generate ATP, necessary for sperm motility [64]. GAPDHS binds to the fibrous sheath of the cytoskeleton surrounding the axoneme in the main part of the sperm flagellum and contains in its active site a highly reactive cysteine residue, essential for its catalytic activity [65,66].

Under pro-oxidant conditions, this cysteine residue can be easily oxidized, resulting in a complete loss of dehydrogenase activity [67]. Although GAPDHS has been identified in sperm from bulls with high fertility [68], Liu et al. [69] associated the inhibition of this enzyme with low percentages of sperm motility in individuals with diabetes mellitus, attributable to oxidative stress caused by the disease. In addition, a positive correlation has been found between a decrease in GAPDHS activity and a reduction in motility in normokinetic spermatozoa incubated with hydrogen peroxide [70].

The identification of GAPDHS in greater abundance in the first ejaculate raises an important paradox: while this enzyme is essential for sperm ATP production and motility, its functionality may have been compromised by oxidative stress. Under pro-oxidant conditions, key cysteine residues in GAPDHS are susceptible to oxidation, leading to enzymatic inactivation and impaired sperm motility. This mechanism has been previously associated with male infertility in various species. This could explain why its high abundance did not translate into higher percentages of sperm motility. However, this hypothesis requires further experimental validation.

Likewise, angiotensin-converting enzyme (ACE) was identified as a DAP in the seminal plasma of the first ejaculate. ACE is a zinc-dependent metalloenzyme that acts as a dipeptidase, playing a crucial role in hormonal signaling pathways [71]. In the case of bovine ACE, its function goes beyond the regulation of hormonal peptides, also participating in the modulation of mRNA stability and contributing to the post-transcriptional regulation of gene expression [72,73]. In the reproductive field, ACE has been associated with low sperm motility in humans and goats [74,75]. In bulls, previous studies have identified ACE as differentially abundant in the seminal plasma of Bos indicus bulls with low sperm motility [76]. These findings are consistent with the results obtained in our study, suggesting that ACE could play a relevant role in the sperm quality alterations associated with oxidative stress and other adverse conditions.

Seminal plasma protein PDC-109 (BSP1) and Epididymal sperm binding protein 1 (ELSPBP1) were identified as DAPs in the first ejaculate. Both are constitutive proteins of bull seminal plasma and contain a fibronectin type 2 domain, which provides them with an affinity for the choline groups of sperm membrane phospholipids [77,78]. The binding of BSP1 and ELSPBP1 to spermatozoa provides stability to the membrane during sperm transit through the female reproductive system and helps in the establishment of the oviductal sperm reservoir [79,80]. Additionally, BSP1 interacts with high-density lipoproteins and heparin-like glycosaminoglycans in the oviductal fluid to orchestrate the initiation and progression of sperm capacitation [77,81]. Despite these characteristics, several studies suggest that the beneficial effects of BSP1 and ELSPBP1 are concentration-dependent [81]. Indeed, BSP1 was identified as the most abundant protein in low-fertility bulls and in subpopulations of immotile bovine spermatozoa, while ELSPBP1 was associated with dead spermatozoa in the cauda epididymis [82,83,84,85].

On the other hand, the Protein LEG1 homolog and Spermadhesin-1 (SPADH1) were identified as DAPs in the last ejaculate. The Protein LEG1 homolog is a protein linked to various biological contexts, performing functions related to lipid biogenesis, intracellular transport, and metabolic homeostasis, depending on the tissue and organism in which it is expressed. Although it has been reported as differentially abundant in spermatozoa and in the seminal plasma of rams, its function in the reproductive context has not yet been fully elucidated [86,87].

For its part, SPADH1 is a multifunctional protein secreted by the accessory sexual glands of the bull, which binds to the phospholipids of the sperm membrane, favoring sperm motility and facilitating passage through the cervical mucus in the female reproductive tract [88,89]. Furthermore, SPADH1 protects sperm from oxidative damage and modulates the endometrial immune response by inhibiting sperm recognition by immune cells [90,91]. This protein has been previously reported as differentially abundant in seminal plasma and sperm from Nellore bulls with high sperm quality parameters [20].

The identification of SPADH1 as a DAP in the seminal plasma of the last ejaculate is consistent with the sperm analyses performed, which showed significantly better parameters compared to the first ejaculate. These results reinforce the influence of SR in the modulation of the proteomic profile of seminal plasma and highlight the importance of key proteins such as SPADH1 in seminal quality and the processes that support fertility in bulls.

In conclusion, our findings suggest that sexual rest modulates the proteomic profile and oxidative status of seminal plasma, affecting sperm quality and the functionality of key proteins in the sperm physiology. The association between the prolonged storage of spermatozoa in extragonadal reserves, oxidative stress, and the activation of apoptotic pathways highlights the importance of considering the depletion of these reserves at the beginning of reproductive seasons in natural mating systems as a strategy to optimize the reproductive management of bulls. Furthermore, we highlight that a single semen collection may not be sufficient to accurately assess the true reproductive potential of a bull, since individual variations can significantly influence the results. Therefore, an adequate diagnosis should be based on an individualized approach for each animal. Likewise, the identification of DAPs with essential functions in sperm motility and viability suggests their potential as biomarkers in the selection of breeding stock. Although this study provides a detailed characterization of the proteomic alterations induced by sexual rest in seminal plasma, future studies should focus on the experimental validation of these differentially abundant proteins to confirm their functional role in bull fertility.

## 4. Materials and Methods

### 4.1. Ethical Approval

The experimental protocol for this study was approved by the Animal Use Ethics Committee of the Universidade Federal de Viçosa under process number 60 of 2019. Additionally, all procedures were performed in strict compliance with the relevant guidelines and regulations, and the methodology was reported in accordance with ARRIVE guidelines (https://arriveguidelines.org, accessed on 20 May 2019).

### 4.2. Animal Selection

This study included six adult Nellore bulls in SR conditions, with an average age of 8.75 years and an average weight of 963.35 kg. All bulls were healthy and were selected based on the results of a Breeding Soundness Evaluation, undergoing sequential semen collections using the electroejaculation method. Semen quality was assessed according to the standards established by the CBRA, ensuring that all the bulls met the necessary reproductive criteria [92]. The bulls were kept on tropical pastures (*Brachiaria brizantha*) and remained sexually inactive for 195 days, during which time they had no contact with females, and no semen collection was performed. All semen collections were conducted on the same day, with 10 min intervals between collections to minimize the risk of injuries resulting from the procedure. During the Breeding Soundness Evaluation, the reproductive organs were examined, and the scrotal circumference was measured at the widest part of the scrotum using a tape measure [93].

After each collection, the volume of the ejaculate (mL) and the physical aspect of the semen (classified from 1 to 4—1 for aqueous, 2 for opalescent, 3 for milky, and 4 for creamy) were determined. The same technician subjectively assessed the physical characteristics of the semen, including mass motility (scored from 0 to 5, where 0 represents no mass motility and 5 indicates marked mass motility), sperm motility (percentage from 0% to 100%, where 0% indicates no motility and 100% indicates full motility), and sperm vigor (scored from 0 to 5, where 0 indicates no movement and 5 indicates rapid and vigorous movement). Assessments were performed using a conventional optical microscope (CX31, Olympus, Tokyo, Japan) at a 100× magnification, and the results were calculated from the average of four microscopic fields. Sperm morphology was analyzed on a wet-stained slide and coverslip at a 1000× magnification using phase-contrast microscopy (BX41, Olympus, Tokyo, Japan) [94]. For each sample, 400 spermatozoa were counted to determine the percentage of spermatozoa with defects in the acrosome, head, intermediate piece, or tail. Defects were classified into three categories, major (associated with reduced fertility), minor (abnormalities of minor importance), and total defects (sum of major and minor defects), according to the criteria described by Blom [95] and recommended by the CBRA [92].

After semen evaluation, the ejaculates were centrifuged at 10,000× *g* for 10 min (Heraeus Multifuges X1R, Thermo Scientific, Waltham, MA, USA) to separate spermatozoa from seminal plasma. Seminal plasma from the first and last ejaculates of each bull was stored in 0.5 mL straws and cryopreserved in liquid nitrogen at −196 °C until later analysis. Subsequently, the seminal plasma was thawed and filtered through a 0.22 µm mesh membrane to yield the supernatant, which was used in the further analysis [20].

### 4.3. Oxidative Stress Sample Preparation

To determine the activity of antioxidant enzymes and the concentration of oxidative markers, aliquots of 200 µL of seminal plasma obtained from the first and last ejaculates of all the bulls were used. Initially, the total protein concentration in each sample was determined using the Bradford method to normalize enzymatic activity. The activity of SOD was evaluated using the pyrogallol auto-oxidation method, as described by Dieterich et al. [96]. CAT activity was determined by measuring the kinetics of hydrogen peroxide decomposition according to the methodology proposed by Aebi [97]. GST activity was analyzed using the method based on the metabolization of 1-chloro-2,4-dinitrobenzene described by Habig et al. [98].

Lipid peroxidation was measured by assessing MDA levels through incubation with thiobarbituric acid, following the protocol for quantifying thiobarbituric acid-reactive substances [99]. Protein oxidation was determined by quantifying CP groups using the 2,4-dinitrophenylhydrazine method, according to Levine et al. [100]. The results were expressed as nmol/mL, using the molar extinction coefficient of ε370 = 22 mmol/L × cm^−1^.

### 4.4. Extraction and Quantification of Soluble Proteins

Soluble proteins were extracted from 100 µL of filtered seminal plasma by precipitation using a solution of ice-cold acetone, trichloroacetic acid (10%, *w*/*v*), and 1 mM dithiothreitol (DTT). The proteins were then solubilized in 250 µL of a solution containing 7 M urea, 2 M thiourea, 4% (*w*/*v*) CHAPS (3-3-cholamidopropyl dimethylammonium-1-propanesulfonate; Sigma-Aldrich, Burlington, NJ, USA), and 40 mM DTT (Sigma-Aldrich, Burlington, NJ, USA). Protein quantification was performed by the Bradford method using a calibration curve generated from known concentrations of bovine serum albumin, ranging from 0.1 to 1.4 mg/mL. Absorbance readings were taken at 595 nm using a spectrophotometer (Multiskan SkyHigth, Thermo Scientific, Waltham, MA, USA) [101].

To verify the quality of the protein extraction and quantification processes, an aliquot of each sample containing 50 µg of soluble proteins was subjected to one-dimensional electrophoresis (SDS-PAGE). The electrophoresis was performed in two steps: protein stacking on a 5.1% T and 2.6% gel, followed by protein separation on a 14% T and 3.0% C gel. The procedure was conducted using the Mini-PROTEAN Tetra Cell system (Bio-Rad, Hercules, CA, USA) at a voltage of 80 V for 15 min, followed by 60 V for 4 h. A broad-range molecular weight marker (Broad Range, Bio-Rad, Hercules, CA, USA) was used. After the run, the gels were stained with Coomassie Brilliant Blue R-250 (Bio-Rad, Hercules, CA, USA) for 2 h and subsequently destained for 8 h in a solution composed of 25% (*v*/*v*) methanol and 7.5% (*v*/*v*) acetic acid [102].

After assessing the quality of the protein extraction and quantification processes by SDS-PAGE, two sample pools were created, composed of seminal plasma from the first and last ejaculates of each bull [20]. Each pool was formed using 100 µg of protein from each sample. Then, five replicates from each pool, containing 50 µg of proteins per replicate, were immobilized and concentrated by SDS-PAGE Short-Run strategy (split: 12.5% T, 3.0% C, stacking: 4.0% T, 2.6% C) using a Mini-PROTEAN Tetra Cell apparatus [103]. The electrophoresis process took place under a constant 80 V, which was stopped when the bromophenol blue indicator reached about 10 mm from the top of the separation gel. After the run, the gels were cut into two parts and placed in a fixative solution (40% (*v*/*v*) methanol and 5% (*v*/*v*) acetic acid) for 2 h. One part of the gel was submitted to the revelation of proteins by the Coomassie Brilliant Blue R-250 method (Bio-Rad, Hercules, CA, USA) for 2 h. The other part of the gel remained in a 5% (*v*/*v*) acetic acid solution for further enzymatic digestion.

### 4.5. Gel Protein Digestion

Each gel fragment was individually excised from the replicates, combined, cut into smaller fragments, and transferred to clean microtubes. The stain was removed from the gels by washing them three times in 200 µL of 50% (*v*/*v*) acetonitrile (ACN) and 25 mM ammonium bicarbonate (pH 8.0). The gel fragments were dehydrated after two incubations with 200 µL of 100% ACN for 5 min and dried in a vacuum concentration system (Concentrator Plus AG-22331, Eppendorf, Hamburg, Germany). The proteins present in the gel fragments were reduced in 100 µL of 65 mM DTT in 100 mM ammonium bicarbonate at 56 °C for 30 min. The proteins were then alkylated with 100 µL of 200 mM iodacetamide and 100 mM ammonium bicarbonate at room temperature for 30 min in the dark. Subsequently, the gel pieces were washed, hydrated, and dehydrated with ammonium bicarbonate and ACN, respectively, twice. Finally, the fragments were dried in a vacuum concentration system.

The dried gel fragments were rehydrated with a trypsin solution (Sigma-Aldrich, Burlington, NJ, USA) at a final concentration of 25 ng/µL in activation solution (40 mM ammonium bicarbonate and 10% (*v*/*v*) ACN). After 45 min in an ice bath, the tubes containing the gel pieces received another 100 µL of the activation solution without trypsin. The tubes were incubated in a water bath at 37 °C for 22 h. After enzymatic digestion, the gels were sonicated for 10 min and vortexed for 20 s, and the resulting solutions were transferred to clean microtubes. Then, 200 µL of the recovery solution (5% (*v*/*v*) formic acid in 50% (*v*/*v*) ACN) was added to the remaining gel fragments. Each microtube containing the gel fragments and the recovery solution was vortexed for 20 s, kept at room temperature for 15 min, and sonicated for 2 min. The solution was removed and added to the previously reserved solution in the clean tube. This procedure was repeated twice, and the final solution was transferred to clean microtubes. The solutions containing the tryptic peptides were concentrated in a vacuum centrifugation system [104]. After vacuum drying, 10 µL of 0.1% (*v*/*v*) trifluoroacetic acid (TFA) solution in ultrapure water was added to each microtube. Then, the tryptic peptides were desalted using C18 reversed-phase microcolumns (Millipore, Burlington, NJ, USA) and eluted in a 50% ACN solution acidified with 0.1% (*v*/*v*) TFA.

### 4.6. Liquid Chromatography–Mass Spectrometry (LC-MS) Analysis

The tryptic peptides from each biological replicate were solubilized in 20 µL of 0.1% (*v*/*v*) aqueous formic acid solution (LC-MS purity grade) and placed in appropriate tubes for application to the nano LC-MS/MS system. After sample preparation, 1 μL of the solution was analyzed by nano LC-MS, using the UPLC nanoAcquity system (Waters, Milford, MA, USA), containing a nanoAcquity UPLC^®^ 2G-V/MTrap 5 µm Symmetry^®^ C18 180 µm × 20 mm trap column, at a flow rate of 7 µL/min, for 3 min. Peptides were separated using a 1.7 µm BEH130 100 µm × 100 mm nanoAcquity UPLC^®^ column, operating at a flow rate of 0.3 µL/min. The mobile phase of the chromatographic process had water acidified with 0.1% formic acid (solvent A) and ACN acidified with 0.1% formic acid (solvent B) as solvents. Chromatographic separation took place according to the following schedule: 2% B for 1 min; gradient from 2 to 30% B for 299 min; gradient from 30 to 85% B for 5 min; maintenance at 85% B for 5 min; gradient from 85 to 2% B for 5 min; and maintenance at 2% B for 5 min, totaling 320 min of chromatographic analysis. The peptides were fed into a MAXIS 3G mass spectrometer (Bruker Daltonics, Billerica, MA, USA) and configured to an online mode using a CaptiveSpray ionization source. An adequate technique (IE GCF 1 February 2017) was used to carry out the analysis of peptides, using a drying gas flow of 3 L/min, an ionization source temperature of 150 °C, and a transmission voltage of 2 kV.

### 4.7. Protein Identification

PEAKS software version 7.0 (Bioinformatics Solutions Inc., Waterloo, ON, Canada) processed the mzXML data. It compared the lists of masses with the Bovidae protein database (Taxonomy ID 9895) deposited in the UniProt Knowledgebase database (UniProtKB, https://www.uniprot.org/, accessed on 3 March 2022). Peptides were then identified using parameters such as methionine oxidation as a variable modification, trypsin enzymatic digestion (with one missed cleavage), and cysteine carbamidomethylation as a fixed modification. The error tolerance for the parent ion was set at 20 ppm and the fragment tolerance at 0.6 Da for the fragments, considering the analysis of ions with charges of +2, +3, and +4. Protein identification was defined by at least two unique peptides with an FDR (False Discovery Rate) lower than 1%.

The label-free quantification analysis considered the peak areas of the proteins identified in the three technical replicates with the highest correlations among themselves per group [13]. The acceptance parameters for the differentially abundant proteins were as follows: a −10 Log P score of 20 (equivalent to a *p*-value of 0.01), a fold change > 2.0, and an FDR < 1% [105]. The label-free quantification and identification of differentially abundant proteins were performed using the proprietary algorithm implementation of PEAKS software version 7.0.

### 4.8. Gene Ontology, Analysis of Functional Clusters, and Protein–Protein Interaction

The gene ontology terms of the identified proteins were obtained from the UniProtKB database using mammalian taxonomy for biological processes, molecular function, and cellular components by the Database for Annotation, Visualization, and Integrated Discovery platform (DAVID; https://david.ncifcrf.gov/home.jsp accessed on 12 January 2023) [106].

### 4.9. Sperm Quality Data Analysis

Differences in sperm quality characteristics among the experimental groups were evaluated using an analysis of variance (ANOVA) with a 5% significance threshold, employing SAEG version 9.1. Oxidative stress analysis data were analyzed using a paired *t*-test, after verifying normality with the Shapiro–Wilk test.

## Data Availability

The data presented in this study are available from the corresponding author upon request.

## Supplementary Materials

The following supporting information can be downloaded at: https://www.mdpi.com/article/10.3390/ijms26062457/s1.

## Abbreviations

The following abbreviations are used in this manuscript:

ACN	Acetonitrile
CAT	Catalase
CP	Carbonyl protein
GDP	Gross Domestic Product
CBRA	Brazilian College of Animal Reproduction
DTT	Dithiothreitol
FDR	False Discovery Rate
GST	Glutathione S-transferase
LC-MS	Liquid chromatography-mass spectrometry
MDA	Malondialdehyde
SOD	Superoxide dismutase
SR	Sexual rest
TFA	Trifluoroacetic acid
