# Supplementary figures and images for: Proteomic Alterations and Oxidative Stress in Seminal Plasma of Nellore Bulls Under Sexual Rest
Source: Int J Mol Sci. 2025 Mar 10;26(6):2457. doi: 10.3390/ijms26062457 (PMC11942078; doi:10.3390/ijms26062457)

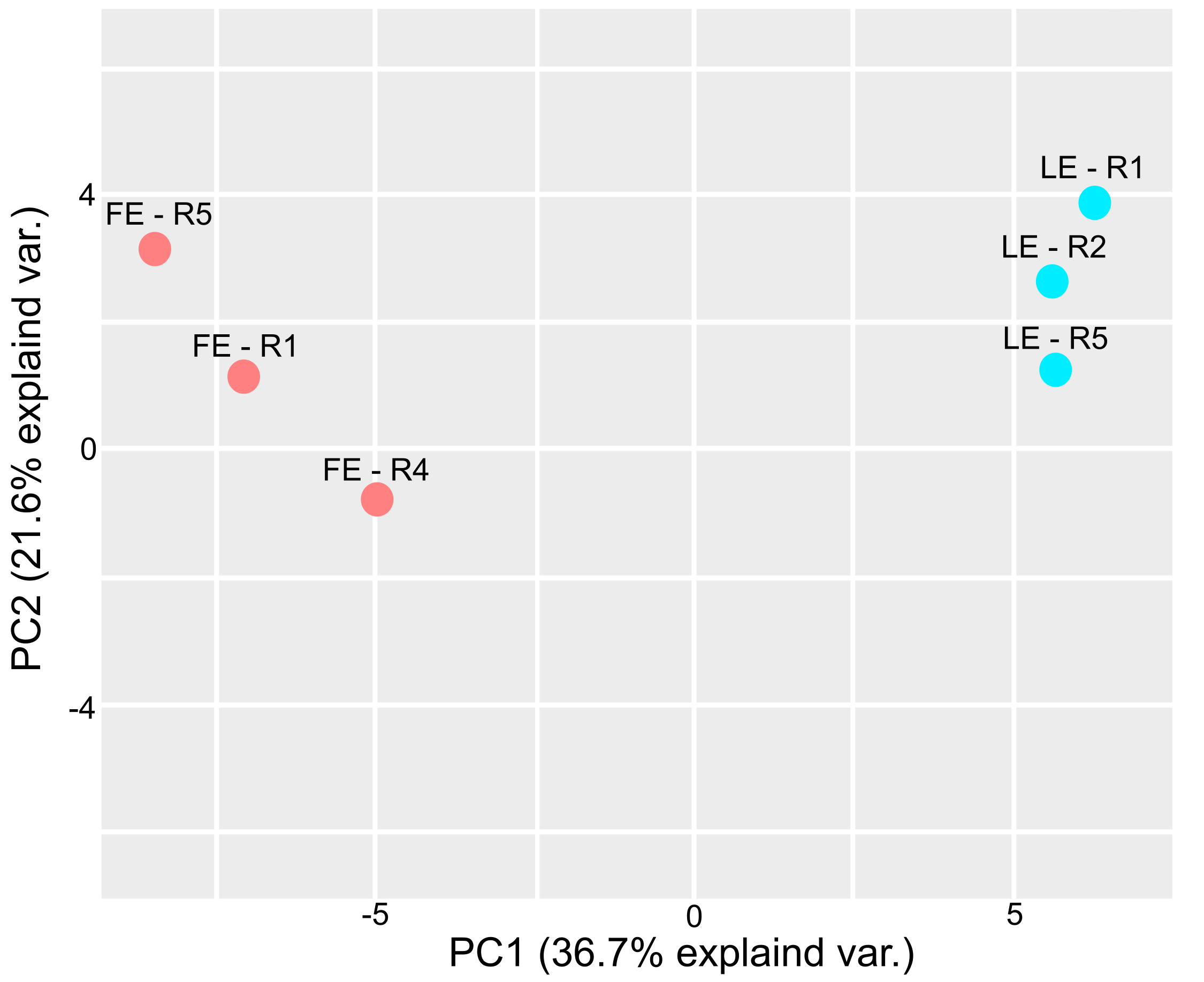

Supplement: Supplementary file 1 [file ijms-26-02457-s001.zip › Figure S1.png]
